# Antiosteoporosis therapy after discontinuation of menopausal hormone therapy: a systematic review

**DOI:** 10.1007/s42000-024-00526-1

**Published:** 2024-01-18

**Authors:** Panagiotis Anagnostis, Efstathios Divaris, Julia Κ. Bosdou, Symeon Tournis, Konstantinos Stathopoulos, Dimitrios G. Goulis

**Affiliations:** 1 Unit of Reproductive Endocrinology, 1st Department of Obstetrics and Gynecology, Medical School, Aristotle University of Thessaloniki, Thessaloniki, Greece; 2 Unit for Human Reproduction, 1st Department of Obstetrics and Gynecology, Aristotle University of Thessaloniki, Thessaloniki, Greece; 3 Laboratory for the Research of Musculoskeletal System “Th. Garofalidis,” School of Medicine, National and Kapodistrian University of Athens, KAT General Hospital, Athens, Greece; 4 School of Medicine, Post Graduate Course on Bone Metabolic Diseases, National and Kapodistrian University of Athens, Mikras Asias 75, 11527 Athens, Greece

**Keywords:** Estrogen, Menopausal hormone therapy, Postmenopausal women, Bisphosphonates, Alendronate, Raloxifene

## Abstract

**Objective:**

Menopausal hormone therapy (MHT) has consistently shown a bone protective effect by reducing the risk of vertebral, non-vertebral, and hip fractures in postmenopausal women regardless of baseline fracture risk. However, the optimal sequential treatment after MHT discontinuation has not been determined. This systematic review aimed to obtain the best evidence regarding the effect of antiresorptive or osteoanabolic treatment on bone mineral density (BMD) and/or fracture risk following MHT.

**Methods:**

A comprehensive search was conducted in the PubMed, Scopus, and Cochrane databases up to October 31, 2023. Randomized-controlled trials (RCTs) and observational studies conducted in postmenopausal women were included.

**Results:**

After the exclusion of duplicates, 717 studies were identified. Two were eligible for qualitative analysis, one RCT and one retrospective cohort study. The RCT showed that alendronate 10 mg/day for 12 months further increased lumbar spine (LS) BMD by 2.3% following MHT and maintained femoral neck (FN) BMD in postmenopausal women (n = 144). It also decreased bone anabolic and resorption markers by 47 and 36%, respectively. In the retrospective study (n = 34), raloxifene 60 mg/day increased both LS and FN BMD at 12 months by 3 and 2.9%, respectively. No fractures were reported.

**Conclusions:**

Antiresorptive therapy with either a bisphosphonate (i.e., alendronate) or raloxifene could be considered a sequential antiosteoporosis therapy after MHT withdrawal since they have been shown in studies to further increase BMD. However, no safe conclusions can be drawn from the existing literature.

## Introduction

Estrogen exerts a beneficial role in bone metabolism, mainly indirectly by preventing bone resorption via estrogen receptor (ER) β through the osteoprotegerin (OPG)/receptor activator of the NF-κB (RANK)/RANK ligand (RANKL) axis [1]. Estrogens also foster osteoblastic activity and differentiation, acting directly through the Wnt pathway, activated by ERα [1]. They also inhibit osteoblast and osteocyte apoptosis through decreased expression of Fas ligand and protein semaphorin 3A, respectively [1]. Estrogen deficiency during and after transition to menopause leads to a progressive decrease in bone mineral density (BMD), predisposing to increased fracture risk, which is mostly evident in women with an early age at menopause [2].

Randomized controlled trials (RCTs) [3] and meta-analyses [4] have consistently shown that menopausal hormone therapy (MHT) is efficacious in reducing the risk of vertebral, non-vertebral, and hip fractures, irrespective of age, falls risk, or baseline FRAX probability [5]. However, when MHT is discontinued, rapid bone loss occurs, mostly within the first 2 years, identical to that seen within the first 2 years of menopause (1.5—2% per year) [6]. The optimal sequential antiosteoporotic treatment after MHT discontinuation has not yet been determimed.

This study aimed to systematically review and meta-analyze the existing evidence regarding the effect of available antiosteoporotic therapies after cessation of MHT to define the optimal sequential treatment.

## Materials and methods

### Guidelines followed

**Fig. 1:**
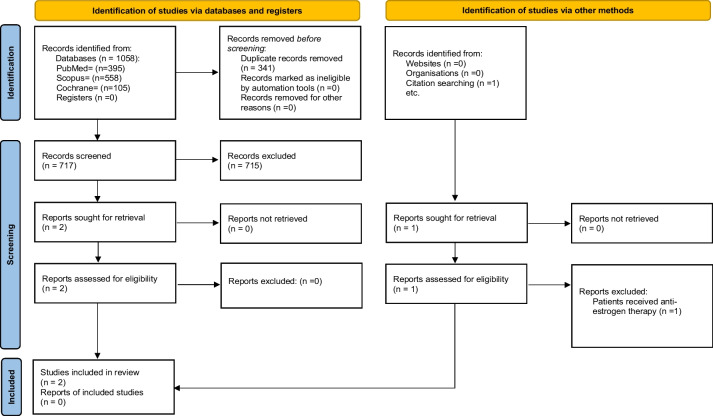
Flow chart diagram

This systematic review followed the Preferred Reporting Items for Systematic Reviews and Meta-Analyses (PRISMA) guidelines [7]. A flow chart diagram is displayed in Fig. 1. The present study has also been registered in the Prospective Register of Systematic Reviews (PROSPERO) System (PROSPERO ID: CRD42023438865).

### Search strategy

A systematic literature search was conducted from conception until July 31, 2023, in the MEDLINE (PubMed), Scopus, and Cochrane databases to identify eligible studies. The set of relevant terms is presented in Supplementary Table 1.

This study was conducted by the PICO (Population, Intervention or exposure, Comparison, Outcome) model for clinical questions, as follows: (i) Population: postmenopausal women who had received MHT for at least 1 year and had low BMD at baseline (i.e., manifested either as osteopenia or osteoporosis); (ii) Intervention: antiosteoporosis (either antiresorptive or osteoanabolic) therapy administered immediately after discontinuation of MHT; (iii) Comparison group: placebo or calcium plus vitamin D (CaD) or no therapy after MHT withdrawal; and (iv) Outcome: percentage (%) or absolute change in BMD or incidence of new fragility fractures. Grey literature was searched using relevant websites. A manual search was also conducted through citation searching of reviews, which were identified by the above systematic search. The main search was completed independently by two investigators (ED and JKB) who checked all available articles.

### Study selection

The specific inclusion criteria were the following: (i) studies conducted in postmenopausal women (either hysterectomized or non-hysterectomized) who had received MHT for ≥ 1 year and had low BMD at baseline, and (ii) studies providing extractable data on BMD or fragility fractures. RCTs and observational studies published in English were eligible. Only studies with a follow-up time of $\ge$ 6 months were included. There was no limitation regarding the year of publication, population enrolled, or patients’ age.

Studies were excluded as follows: if they (i) were conducted in patients receiving therapy associated with bone loss, such as aromatase inhibitors or glucocorticoids; (ii) had included patients with metabolic bone diseases (e.g., rheumatoid arthritis or Paget’s disease) or secondary causes of osteoporosis (e.g., primary or secondary hyperparathyroidism, osteomalacia, thyrotoxicosis, Cushing’s syndrome, malabsorption syndrome, diabetes mellitus, rheumatoid arthritis, or genetic causes of osteoporosis); (iii) were written in a non-English language, (iv) were conducted in animals; and (v) were studies not answering the research question.

### Data extraction

The following data were extracted and recorded: (i) first author’s name, (ii) year of publication, (iii) study design, (iv) country in which the study was conducted, (v) number and mean [± standard deviation (SD)] age of participants, (vi) duration of MHT, (vii) duration of antiosteoporosis treatment, (viii) BMD before and after antiosteoporosis therapy, and (ix) incidence of fragility fractures during treatment. Parameters, such as mean (± SD) body mass index (BMI), age at menopause, smoking status, alcohol intake, and physical activity, were also recorded where available.

The following comparisons were made: (i) absolute (in g/cm^2^) or percentage (%) change in BMD in women who received antiosteoporosis therapy after MHT discontinuation compared with those who received placebo, CaD, or no treatment, and (ii) fracture incidence in women who received antiosteoporosis therapy after MHT discontinuation compared with those who received placebo, CaD, or no treatment.

### Risk of bias and study quality assessment

Risk of bias assessment for RCTs was carried out using *Review Manager* (*RevMan* computer program), version 5.4.2 software (Copenhagen: The Nordic Cochrane Centre, The Cochrane Collaboration, 2014), whereas the Newcastle–Ottawa scale (NOS) was used to assess the quality of observational studies. This scale evaluates studies according to three criteria, as follows: (i) participant selection, (ii) comparability of study groups, and (iii) assessment of outcome or exposure. Each category is assessed based on a four, two, and three-star scale, respectively. A study is characterized as “good quality” when it gets 3–4 stars in the selection domain, 1–2 stars in the comparability domain, and 2–3 stars in the outcome/exposure domain. “Fair quality” is considered to apply when two stars are obtained in the selection domain, 1–2 stars in the comparability domain, and 2–3 stars in the outcome/exposure domain. Finally, a study is characterized as being of “poor quality” in the case of 0–1 stars in the selection domain or 0 stars in the comparability domain or 0–1 stars in the outcome/exposure domain [8].

## Results

### Descriptive data

The initial research yielded 717 articles, after excluding duplicates, two of which were assessed as full-text papers [9, 10]. In addition, reference searching of the selected studies provided another article [11], which was excluded due to the use of antiestrogen therapy by all study participants. These two studies [9, 10] were also included in the qualitative analysis. The 715 studies were excluded by title and/abstract mainly because they did not answer the research question. Other reasons were the inclusion of patients under treatment with medication which are associated with increased bone loss, or patients with secondary causes of osteoporosis. The flowchart diagram is presented in Fig. 1. The main characteristics of the studies and their participants are presented in Table 1. All postmenopausal women had received MHT for at least 1 year.

**Table 1:** Demographic characteristics of studies included in the analysis

ID	Author/ year of publication	Study Design	Country	Total number	Anti-osteoporosis therapy	Mean age (years) ± SD	Mean BMI (kg/m^2^) ± SD	Years post-menopause	LS T-score (SD)	Outcome assessment
1	Evans, 2003	RCT	Multicenter	144	Alendronate 10 mg/day for 12 months	57.3 ± 6.6	24.8 ± 3.4	11.5 ± 7.3	−2.27 (0.65)	• LS, FN, hip trochanter, total body BMD • Bone turnover markers
2	Song, 2006	Retrospective cohort	South Korea	34	Raloxifene 60 mg/day for 12 months	59.2 ± 7.6	22.5 ± 2.1	10.3 ± 7.81	−2.08 (0.69)	• LS, FN BMD • Vertebral fractures

Abbreviations: *BMD* bone mineral density; *FN* femoral neck; *LS* lumbar spine; *MHT* menopause hormone therapy; *RCT* randomized-controlled trial; *SD* standard deviation

The first study was an RCT, published in 2003, which evaluated the effect of alendronate 10 mg/day plus calcium 500 mg/day, in comparison with placebo, on BMD at the lumbar spine (LS), femoral neck (FN), hip trochanter (HT), and total body (TB) in 144 postmenopausal women, after 12 months of treatment (mean LS BMD T-score at baseline: -2.27 ± 0.65). These women had received MHT for ≥ 1 year and were enrolled within 3 months after MHT discontinuation. The effect on bone turnover markers (BTM), in particular, serum bone-specific alkaline phosphatase (BALP) and urinary N-telopeptide of type I collagen corrected for creatinine (Ur NTx/Cr), was also assessed [9]. Alendronate further increased LS BMD by 2.3% following MHT, whereas a mean loss of 3.2% was observed in the placebo group (mean difference: + 5.5% between groups). Regarding FN, alendronate maintained BMD, whereas it decreased by 1.4% in the placebo group [9]. BALP and Ur NTx/Cr decreased by 47 and 36%, respectively, in the alendronate group, whereas they increased by 20 and 36%, respectively, in the placebo group. Mean changes with confidence intervals (CIs) of BMD and BTM are presented in detail in Table 2. Alendronate was well-tolerated. Hot flushes were reported in 16% after MHT withdrawal [9].

**Table 2:** Results of studies included in the review

ID	Study	LS BMD change at 12 months	FN BMD change at 12 months	BALP change at 12 months	Ur NTx/Cr change at 12 months	Vertebral fractures
1	Ascott Evans, 2003					
	*Alendronate*	2.3% (95% CI 1.7% to 3.0%)	0.2% (95% CI -0.6 to 1.0%)	−19.6% (95% CI -26.7 to -11.8%)	−46.7% (95% CI -55.6% to -36.2%)	N/A
	*Placebo*	−3.2% (95% CI -4.6% to -1.7%)	−1.4% (95% CI -2.3 to −0.4%)	17.9% (95% CI 1.8% to 36.6%)	35.7% (95% CI 6.7% to 72.4%)	
2	Song, 2006					
	*Raloxifene*	2.9% (± 4.6%)	3% (± 6.6%)	N/A	N/A	0%

Abbreviations: *BMD* bone mass density; *BALP* bone-specific alkaline phosphatase; *CI* confidence interval; *FN* femoral neck; *N/A* not available; Ur NTX/Cr: urinary N-telopeptide of type I collagen corrected for creatinine

The other study assessed, retrospectively, the effect of 12-month treatment with raloxifene 60 mg/day plus calcium 500 mg/day on LS and FN BMD as well as the incidence of vertebral fractures in 34 postmenopausal women, who had received MHT for ≥ 1 year and had discontinued it within a month before raloxifene therapy in initiation. Mean LS and FN BMD T-scores at baseline were -2.08 ± 0.69 and -1.71 ± 0.50, respectively. Both LS and FN BMD increased by 3 and 2.9% respectively. No fractures were reported. A limitation of this study was the lack of a placebo group [10]. Mean changes with confidence intervals (CIs) of BMD and BTM are presented in detail in Table 2.

Regarding risk-of-bias, the study by Ascott-Evans et al. was considered as being of “low risk of bias” [9], whereas the study by Song et al. was of “poor quality” [10].

### Meta-analysis

Meta-analysis could not be performed due to the different type of treatment and study design between the two studies.

## Discussion

The present study provides evidence for sequential antiresorptive therapy after MHT withdrawal. Only two studies are currently available, both showing a beneficial effect for LS and FN BMD, with either alendronate or raloxifene, after completion of 12 months with MHT.

MHT is one of the most efficacious antiosteoporotic therapies since it reduces all types of fractures, regardless of baseline BMD [12]. According to the most representative RCT, the Women’s Health Initiative (WHI), MHT consisting of conjugated equine estrogen (CEE) 0.625 mg/day and medroxyprogesterone acetate 2.5 mg/day (n = 8,506) compared with placebo (8,102) reduces the risk of total, vertebral, hip, and wrist fractures by 24, 35, 33 and 29%, respectively [3]. This effect is independent of age, time since menopause, progestin use, BMI, smoking, number of falls, and total calcium intake [3] as well as of FRAX score [5]. It must be highlighted that MHT is the most effective and strongly evidence-based therapy for women at low fracture risk, such as those aged < 60 years old. One must remember that there is no evidence for anti-fracture benefits in this age group with the commonly used medications, such as bisphosphonates, teriparatide, and denosumab, as shown in their historical RCTs. The mean participants’ age in all these hallmark studies was > 65–70 years [13–16]. However, except for cases with early menopause or POI, MHT is indicated in women with vasomotor symptomatology and younger than 60 years of age or with 10 years since their last menstrual period, and not for the sole purpose of osteoporosis management [17].

Observational data have shown that BMD progressively decreases after MHT discontinuation (1.5–2% annually) [6], although it remains above the pre-treatment levels. Some studies have shown higher rates of bone loss (i.e., -7.69% in LS and -5.16% in total hip) in women who had received MHT for 4 years, during 12–24 months after treatment cessation [18]. However, a more recent study showed that the beneficial effect of MHT on BMD and bone microarchitecture persists after cessation of MHT [19]. Fracture risk does not seem to increase in the short term after MHT discontinuation [1]. Post-hoc analysis of the WHI trial showed no evidence of increased fracture risk after MHT cessation [20]. Moreover, in a prospective cohort study (n = 263 postmenopausal women), BMD remained higher in those women who received 2 mg 17β-estradiol/day for ≥ 2 years compared with placebo, even 5–15 years after MHT withdrawal. The latter BMD maintenance was associated with a reduction in the risk of major osteoporotic and vertebral fractures [odds ratio (OR) 0.48 (95% CI 0.26–0.88) and 0.47 (95% CI 0.24–0.9), respectively] [21].

In general, there are currently no formal guidelines concerning the optimal sequential therapy after MHT discontinuation. These two studies, which were included in the present systematic review [9, 10], provide evidence that in cases with a T-score of -2 at the time of MHT cessation, using either alendronate or raloxifene further increases BMD, especially at the LS. In any case, switching to an antiresorptive (i.e., bisphosphonates, denosumab, or raloxifene), osteoanabolic (i.e., teriparatide, abaloparatide), or even a dual-acting agent (i.e., romosozumab, in cases of high or very high risk), depending on the woman’s residual fracture risk, is prudent as a BMD-maintenance policy after MHT withdrawal [17].

The main strength of the present study is that it constitutes, to the best of our knowledge, the first systematic review regarding the optimal sequential antiosteoporotic regimen in postmenopausal women after MHT withdrawal. Limitations include the small number of studies, the small sample size, and the heterogeneity regarding the antiosteoporotic agent used and the study design. The lack of comparative data between the available therapies constitutes another limitation.

To summarize, no safe conclusions can be drawn from the existing literature regarding the optimal sequential antiosteoporosis therapy after MHT discontinuation. However, alendronate or raloxifene may be considered for at least 12 months to prevent potential bone loss. Therefore, there is an exigent need for future RCTs to assess the effect of different therapeutic interventions for BMD and fracture risk in these cases.
